# Delving into discrepancies, a single-center experience with Accelerate Pheno for gram-negative bacteremia, a rapid phenotypic susceptibility testing method

**DOI:** 10.1017/ash.2024.482

**Published:** 2025-01-23

**Authors:** Zoe Freeman Weiss, Dimitar Zelenkov, Jon Englert, Maureen Campion

**Affiliations:** 1 Department of Pathology, Tufts Medical Center, Boston, MA, USA; 2 Division of Geographic Medicine and Infectious Diseases, Tufts Medical Center, Boston, MA, USA; 3 Tufts Medical Center, Department of Pharmacy, Boston, MA, USA; 4 Accelerate Diagnostics, Tucson, AZ, USA

## Abstract

**Objective:**

Discrepancies or inaccuracies between testing methods can create confusion or lead to clinical harm if antibiotics are inappropriately chosen. We report our clinical experience using the Accelerate Pheno™ followed by routine automated susceptibilities by the Vitek®2 for positive blood cultures with gram-negative rods.

**Design:**

This was a retrospective review of positive gram-negative blood cultures, including comparison of susceptibility testing results and impact on clinical care.

**Setting:**

Academic teaching hospital.

**Patients (participants):**

All patients admitted to the hospital with gram-negative bacteremia from January 2020 to December 2022.

**Methods:**

Microbiology was reviewed for discrepancies as defined by very major errors (VMEs), major errors (MEs), and minor errors (mEs). Clinical charts were reviewed for antibiotic therapy.

**Results:**

Positive blood cultures with gram-negative rods were included (n = 262). Between these two methods, overall essential agreement was 93.7% (2162/2304) and categorical agreement 93.5% (2159/2306). There were 147 discrepancies noted, including 6 VMEs, 25 MEs, and 116 mEs accounting for 96 patients. Antibiotic choice was changed in 8 patients due to perceived suboptimal empiric therapy based on the rapid susceptibility results.

**Conclusions:**

The Accelerate Pheno tended to over-call resistance compared to the Vitek®2. Few patients (8) received the incorrect antibiotic based on the Pheno result. Stewardship programs may choose to optimize their rapid antibiotic susceptibility testing reporting to help minimize confusion and guide appropriate antibiotic selection.

## Introduction

There is increasing interest in providing clinicians with rapid phenotypic antibiotic susceptibility testing (AST) directly from positive blood cultures. Rapid AST approaches aim to facilitate earlier antibiotic optimization, whether de-escalation from broad-spectrum empiric therapy or escalation in the case of multidrug-resistant organisms.

Routine susceptibility testing in the clinical microbiology lab typically involves commercial systems for monitoring blood cultures that are incubated in liquid media. When the instrument flags a culture as positive (between 1 and 5 d), it is typically sub-cultured to solid media until visible colonies are formed and then transferred to an automated susceptibility platform. This process may take up to three days from positive blood culture to antibiotic susceptibility finalization.^1,2^ Platforms providing rapid identification and/or susceptibility testing are increasingly being implemented in clinical settings. Some studies have reported reduced turnaround time of susceptibility results,^3,4^ reduced duration of antibiotic use,^5,6^ earlier time to optimal therapy,^7–10^ and reduced hospital length of stay^5^ when implementing rapid AST in a clinical setting. Other studies have not demonstrated appreciable clinical impact.^11^ The level of evidence and robustness of clinical outcome data still necessitates further study.^12,13^ Antibiotic stewardship efforts are recommended for successful implementation of rapid platforms.^14^ Preliminary susceptibility testing would ideally match final susceptibility results. Discrepancies between automated AST platforms are inevitable; however, high rates of discrepancies may lead to clinician mistrust of the technology and thwart early de-escalation efforts.

Our institution uses the Accelerate Pheno™ platform for identification and rapid susceptibility testing of blood cultures positive for gram-negative rods, providing a susceptibility result within 6 hours of blood culture positivity. The Accelerate Pheno™ platform uses morphokinetic cellular analysis (single-cell microscopy) to provide phenotypic susceptibility results directly from positive blood cultures. The Accelerate Pheno™ has demonstrated excellent concordance with existing automated susceptibility platforms and reference broth microdilution methods.^15,16^ Due to the limited number of antimicrobials on Accelerate Pheno™ and desire to maintain consistency in AST methodology across the laboratory, susceptibilities are still performed using the Vitek®2, which is used at our institution for the majority of clinically significant isolates from all sources. Final Vitek®2 results are subsequently available 48–72 hours after preliminary susceptibilities are available. Of note, the Vitek®2 is a modified broth microdilution method that extrapolates minimum inhibitory concentration (MIC) values based on continuous growth curve analysis, comparing the test isolate growth curves to reference isolate growth curves with known MICs. This platform is FDA-approved for AST with sufficient concordance to gold standard agar diffusion or broth microdilution methods to allow for routine clinical use and reporting.^17^

Data indicate that the greatest benefit from rapid AST platforms is in conjunction with stewardship interventions.^18–20^ Our antibiotic stewardship team is alerted when results of rapid susceptibility testing are available to help support primary teams in adjusting antibiotics when appropriate. Occasionally, discrepancies between the rapid AST method and Vitek®2 are observed. We aimed to evaluate the frequency and clinical impact of discordance between rapid susceptibility testing and routine standard of care. We discuss a practical approach to the use of rapid susceptibility testing in a clinical setting.

## Methods

We retrospectively reviewed all positive blood cultures with gram-negative rods at Tufts Medical Center, a 415 bed academic tertiary care center over a 2-year period between January 2020 and December 2022. We compared the results of the Accelerate Pheno™ platform (software version 1.4.1.28–1.5.0.30, Accelerate Diagnostics, Inc., Tucson, AZ) with the Vitek®2 (BioMérieux, Marcy-l’Étoile, France). Software for the Accelerate Pheno™ platform was updated to 1.5.0.30 in January 2021 which included a reformulation of *Pseudomonas aeruginosa* AST assays for ceftazidime, cefepime, piperacillin-tazobactam, and meropenem. As such, results for these antibiotics and *Pseudomonas aeruginosa* prior to this software update were not included in performance analysis.

Testing on the Vitek®2 was performed using the FDA-cleared Vitek®2 system workflow. Exclusion criteria for AST analysis included off panel organisms, polymicrobial samples, instrument failure, and incorrect/mismatched ID results. Because our institution routinely uses the Vitek®2 for the majority of clinical isolates, we used this as the reference method. This study was reviewed and approved by the Tufts University Institutional Review Board (Study 00003698).

We quantified the frequency of minor, major, and very major discrepancies and reported essential and categorical agreement. Essential agreement refers to the percent of test MICs that are within +/- 1 doubling dilution of the reference method, while categorical agreement refers to the % of test results within the same category of result as the reference method (susceptible/intermediate/resistant). Minor errors (mEs) are instances where an isolate is reported to be intermediate to a drug by the test method but was either susceptible or resistant by reference method. Major errors (MEs) are defined as an isolate determined to be resistant by the test method but sensitive by the reference method. Very major errors (VMEs) are defined as when an isolate is sensitive to a drug by the test method and resistant by the reference method. The rate of VME is defined as the number of VME divided by the number of resistant samples, while the ME rate is defined as the number of ME divided by the number of susceptible samples. No significant changes in susceptibility breakpoints for the antibiotics reported occurred within the study period.

For all instances of discrepant results, we reviewed the clinical charts to evaluate the impact of discrepant results on antibiotic choice. We determine whether or not therapy was altered in the presence of discrepant susceptibility testing.

## Results

**Figure 1.**
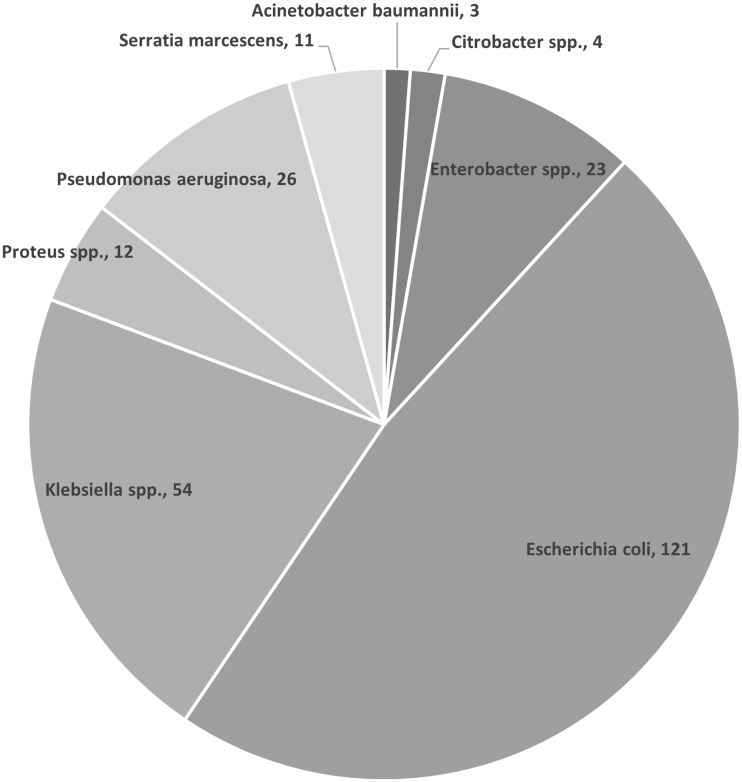
Distribution of gram-negative species identified by Accelerate Pheno™.

A total of 262 positive aerobic blood cultures with gram-negative rods were identified over the study period. The distribution of species is shown in Figure 1. There were 237 samples eligible for susceptibility testing: 1 was excluded due to an identification mismatch, 4 did not have secondary AST available, and 6 were excluded due to growth control failure. Overall, 226 samples were included in the primary analysis. Essential agreement was 93.7% and categorical agreement 93.5%.

Including available discrepancy adjudication, there was a 2.8% (6/220) VME rate, 1.3% (25/2051) ME, and 5% mE rate (116/2306). Of the mEs 90.5% (105/116) overcalled resistance by Accelerate Pheno™ compared to the VITEK2 (Table 1). Of note 3/6 VMEs were due to ciprofloxacin. Essential and categorical agreement were lowest in ceftazidime (77.2% and 79.5%, respectively). Discrepancy resolution was only performed in 16 instances of ME or VME. In 4 instances, broth microdilution confirmed the results of the Accelerate Pheno™ (1 VME, 3 ME), 10 isolates confirmed the results of the Vitek (10 ME), and 2 were intermediate (2 ME).

**Table 1.** Essential and categorical agreement between the Pheno and VITEK 2 system using VITEK 2 as a reference method

Antibiotic	Count	Essential agreement	Count	Categorical agreement	Very Major discrepancies	Major discrepancies	Minor discrepancies
Amikacin	222/222	100%	221/222	99.5%	0	0	1
Gentamicin	210/223	94.2%	220/223	98.7%	0	0	3
Tobramycin	215/220	97.7%	214/220	97.3%	0	0	6
Ceftazidime	169/219	77.2%	174/219	79.5%	0	11^1^	33
Cefepime	200/224	89.3%	198/224	88.4%	1	6^2^	15
Ceftriaxone	198/200	99%	190/200	95%	1	1^3^	8
Ertapenem	199/199	100%	198/200	99%	0	0	2
Meropenem	201/211	95.3%	206/211	97.6%	0	1^4^	2
Ampicillin-Sulbactam	160/173	92.5%	149/173	86.1%	0	1^5^	23
Piperacillin-tazobactam	180/204	88.2%	182/205	88.8%	1^6^	4^7^	18
Ciprofloxacin	206/212	97.2%	202/212	95.3%	3	2^8^	5
Total:	2160/2307	93.6%	2154/2309	93.3%	6	25	116

1 Six ME were adjudicated with broth microdilution testing. Five agreed with Vitek®2 and one was changed to a minor error.

2 Three ME were adjudicated with broth microdilution testing. Three agreed with Accelerate Pheno™.

3 One ME was adjudicated with broth microdilution testing and agreed with Vitek®2.

4 One ME was adjudicated with broth microdilution testing and agreed with Vitek®2.

5 One ME was adjudicated with broth microdilution testing and was changed to minor error.

6 One VME was adjudicated with broth microdilution testing and agreed with Accelerate Pheno™.

7 Two ME were adjudicated with broth microdilution testing. Two agreed with Vitek®2.

8 One VME was adjudicated with broth microdilution testing and agreed with Vitek®2.

The total discrepancy number (N = 147) accounted for 96 distinct patients. Due to changes in the electronic medical record and data recording practices 7 were excluded. Eighty-nine patients had AST discrepancies that were evaluated for clinical impact by chart review. In 81/89 (91%) discordant AST results did not impact antibiotic choice and had no clinical impact. In 8/89 (9%) patients, antibiotic discordance affected selection of antibiotic therapy, accounting for 6 mEs, 1 ME, and 1 VME.

## Discussion

There is very little published data exploring the real-world clinical implications of discrepancies identified in a clinical microbiology laboratory that employs a rapid commercial AST platform alongside routine susceptibility testing.^11^ Even with highly accurate FDA-approved platforms, occasional discrepancies between AST methods are to be expected when performing duplicate testing on the same organism. This is especially true for some broad-spectrum antibiotics such as piperacillin-tazobactam, which has been historically difficult to perform accurate and reproducible AST even by gold standard methods.^21^ We report high concordance between the Accelerate Pheno™ and the automated Vitek®2 AST results in a real-world setting. Overall categorical and essential agreement were >90%, similar to other published data.^15,22,23^ Though discrepancies were uncommon overall, they can lead to changes to antibiotic therapy, provider confusion, or potential distrust of results. Similar to previously published studies, we note that the majority of discrepancies (88.4%) tended to overcall resistance compared to the Vitek®2.^6,23–25^ From a patient safety perspective, rapid susceptibility methods that may overcall resistance are generally preferred to those that potentially under-estimate resistance, given the risk of under treatment in the latter.

Robinson et al (2021) reviewed all gram-negative blood cultures and compared clinical impact of discordance between standard of care to Pheno testing in a subset of cultures. They cited a higher number of patients having a prescribing impact based on discrepancies, 55%, with the most common being continuation of overly broad therapy.^11^ In our study, discordance leading to inappropriate changes in antimicrobials were infrequent (9%). However, conflicting results and the need to change antimicrobial therapy multiple times can lead to clinician mistrust of results in the future. Some infectious disease physicians documented their preference to “wait for final susceptibilities” before changing antibiotics. Therefore, additional education with antimicrobial stewardship groups and published discrepancy analyses are helpful to show the low incidence of discordance, the nature of common discordant results, and outline the laboratory and clinical approach to adjudication. Microbiology laboratories performing testing should maintain technical reporting guidelines for testing personnel, indicating when to suppress antibiotics, when to perform discrepancy testing, when to consult with an on-call clinical microbiologist, and how to report results.^6^

For example, given that ceftazidime is not used as a primary empiric agent at our institution (cefepime or ceftriaxone are favored) and the high rate of ME and mE for this antibiotic, we suppressed the results of ceftazidime from future rapid AST reporting. We felt that the “overcalling” of ceftazidime resistance, even when ceftriaxone susceptible, influenced clinicians to continue cefepime or meropenem, due to concern for the presence of an extended-spectrum beta-lactamase (ESBL), as shown in cases 1, 2, and 3 (Table 2).

Technicians were instructed to suppress and then repeat testing if Accelerate Pheno™ results did not make sense. For example, if results demonstrated intermediate or resistant results to cefepime, carbapenems, or piperacillin-tazobactam but were reported as susceptible to ceftriaxone or ceftazidime, technicians were asked to suppress these results. Oftentimes, these patterns were not noticed until after results had been released to clinicians, and discrepancies with final AST were already reported. Many laboratories are not fully staffed with clinical microbiologists 24/7, and complex manual reporting/suppression guidelines may be difficult to teach or enforce among staff who have little specialized training in microbiology. Ideally, reporting contingencies should be built into the electronic medical record or with custom rules on the diagnostic device to reduce the probability of errors.

Discrepant AST results may also have important implications for infection control practices. Note that one of the patients in Table 2 was flagged as having an ESBL, leading to changes in their isolation status per infection control, which was later redacted based on the final susceptibilities. If a rapid AST platform overcalls resistance, erroneous patient cohorting may occur occasionally. It is important for hospitals auto-flagging-resistant isolates within their electronic medical records to be aware that these discrepancies can occur and may affect patient contact precautions and isolation status.

**Table 2.** Discordant results for 8 patients whose antibiotics were changed due to discrepancies in susceptibility methods. Only AST results with discrepancies that affected therapy are shown

Patient number	Organism	Antibiotic	Pheno result*	Vitek Result*	Category of error	Impact on antibiotic use
1	Escherichia coli	Ceftazidime Cefepime	I I	S < = 1 S < = 1	Minor Minor	Patient switched from ceftriaxone to carbapenem due to concern for ESBL-producing organism, switched back once Vitek susceptibilities resulted
2	Escherichia coli	Ceftazidime	I	S < = 1	Minor	Patient switched from ceftriaxone to carbapenem due to concern for ESBL-producing organism, switched back once Vitek susceptibilities resulted
3	Klebsiella sp.	Ceftazidime	I	S < = 1	Minor	Neonate on ceftazidime empirically for sepsis and switched to cefepime for full duration of therapy
4	Enterobacter sp	Piperacillin/tazobactam Cefepime	I I	S < = 4 S < = 1	Minor Minor	Started empirically on piperacillin/tazobactam for ascending cholangitis and then switched to ceftriaxone based on susceptibility by Accelerate Pheno™
5	Serratia marcescens	Meropenem Ceftriaxone	I I	S < = 0.25 S < = 1	Minor Minor	Empirically started on meropenem. Based on Accelerate Pheno™ results, gentamicin was added to the regimen, completed therapy on sulfamethoxazole/trimethoprim
6	Escherichia coli	Ceftriaxone	S	R > 64	Very Major	Empirically started on ceftriaxone and was continued until Vitek results showed ceftriaxone resistance and switched to ertapenem to complete therapy
7	Klebsiella sp.	Ceftriaxone	R	S < = 1	Major	Empirically started on cefepime due to ceftriaxone resistance from Accelerate Pheno ™, this was a presumed ESBL and antibiotics changed to ertapenem. Vitek resulted as ceftriaxone susceptible, and patient was then switched to ceftriaxone to complete therapy
8	Enterobacter sp.	Ertapenem	S	I	Minor	Started on ertapenem empirically and continued based on Accelerate Pheno™. Switched to meropenem once Vitek resulted ertapenem intermediate, then completed course on cefepime

S = susceptible, I = intermediate, R = resistant

1. ESBL; extended-spectrum beta-lactamase

This study is subject to a number of limitations. This was a single-site experience at an academic teaching hospital with a well-established antibiotic stewardship program, so results may not be applicable to all institutions. Some antibiotics included in the performance characteristic analysis were not released to clinicians as they were RUO at the time of testing (eg, Ciprofloxacin), so these discrepancies had no potential for clinical impact. Though the Vitek®2 system is one of the most commonly used AST methods in clinical microbiology labs, it is not a true broth microdilution method, instead extrapolating AST results based on growth curves. The Vitek®2 also uses a proprietary advanced expert system^26^ to adjust susceptibility results when unusual phenotypes are produced by testing, which could result in a categorical change, and lead to additional discrepancies. Although we used the Vitek®2 as a comparator, it is not a true gold standard, and discrepancies may have been due to errors in either platform. Tie breaking discrepancy testing was not performed in all cases, which would have helped better characterize the nature of these discrepancies. Future comparison studies are needed to adjudicate methodological accuracy between these two platforms. Although changes in antibiotic therapy were evaluated, clinical outcomes including increased length of stay and mortality were not. Due to the nature of a small retrospective study, this would have been difficult to determine based on antibiotic selection alone.

Though AST is not reported from the Accelerate Pheno™ for polymicrobial samples, there may be instances where the Accelerate Pheno™ identifies an organism and performs susceptibilities, and an additional organism grows later in culture. In these scenarios, antibiotic narrowing may not be appropriate and could lead to undertreatment. Of note, one patient was identified where the Accelerate Pheno™ reported a *Proteus mirabilis* with ceftriaxone susceptibility; however, the final sub-culture grew both an *E. coli* and *Proteus mirabilis.* The *E. coli* was resistant to ceftriaxone. In this case, antibiotics were inappropriately narrowed in the setting of a cryptic polymicrobial infection. Polymicrobial bloodstream infections are uncommon overall, but clinical laboratories should be aware of this limitation.

Implementation of rapid susceptibility testing platforms requires antibiotic stewardship efforts to help clinicians determine the best course of action when discrepancies arise. AST reporting should be tailored to an individual institution’s needs, and laboratories may consider automated reporting rules and selective suppression for non-preferred or non-formulary antibiotics. Discrepancy testing should be performed when possible.

## Conclusion

Accelerate Pheno™ had high concordance with automated Vitek® 2 AST at a large academic medical center. Discrepancies in results leading to inappropriate antibiotic therapy were rare and most commonly lead to overly broad therapy, instead of too narrow of therapy. Stewardship programs can assist in interpreting AST and guiding appropriate antibiotic selection. Further studies on the clinical impact of microbiological discrepancy data should be conducted.

## Supporting information

Freeman Weiss et al. supplementary material Freeman Weiss et al. supplementary material

## Supplementary material

For supplementary material accompanying this paper visit http://doi.org/10.1017/ash.2024.482. click here to view supplementary material
