# Supplementary material for: Delving into discrepancies, a single-center experience with Accelerate Pheno for gram-negative bacteremia, a rapid phenotypic susceptibility testing method
Source: Antimicrob Steward Healthc Epidemiol. 2025 Jan 23;5(1):e15. doi: 10.1017/ash.2024.482 (PMC11795440; doi:10.1017/ash.2024.482)
Supplement: Freeman Weiss et al. supplementary material [file S2732494X24004820sup001.docx]

|  | **Pheno interpretation higher than Vitek** | | **Pheno interpretation lower than Vitek** | |
| --- | --- | --- | --- | --- |
| **Antibiotic** | **Count** | **Relative % of MIN** | **Count** | **Relative % of MIN** |
| Amikacin | 1 | 100% | 0 | 0% |
| Gentamicin | 3 | 100% | 0 | 0% |
| Tobramycin | 3 | 50% | 3 | 50% |
| Ceftazidime | 32 | 96.97% | 1 | 2.94% |
| Cefepime | 14 | 93.33% | 1 | 6.25% |
| Ceftriaxone | 8 | 100% | 0 | 0% |
| Ertapenem | 0 | 0% | 2 | 100% |
| Meropenem | 2 | 100% | 0 | 0% |
| Ampicillin-Sulbactam | 21 | 91.3% | 2 | 8.7% |
| Piperacillin-Tazobactam | 17 | 94.44% | 1 | 5.56% |
| Ciprofloxacin | 4 | 80% | 1 | 20% |
|  | 105 |  | 11 |  |

Supplementary table 1. Breakout of minor errors
